# Geriatric 8 Score Predicts Functional Decline After Endoscopic Resection for Upper Gastrointestinal Neoplasms in Older Adults: A Prospective Cohort Study

**DOI:** 10.1002/deo2.70226

**Published:** 2025-10-18

**Authors:** Yuki Okubo, Takahiro Inoue, Shunsuke Yoshii, Masamichi Arao, Hiroko Nakahira, Taro Iwatsubo, Katsunori Matsueda, Minoru Kato, Satoki Shichijo, Takashi Kanesaka, Sachiko Yamamoto, Koji Higashino, Noriya Uedo, Tomoki Michida, Ryu Ishihara

**Affiliations:** ^1^ Department of Gastrointestinal Oncology Osaka International Cancer Institute Osaka Japan; ^2^ Department of Gastroenterology and Hepatology Kyoto University Graduate School of Medicine Kyoto Japan; ^3^ Second Department of Internal Medicine Osaka Medical and Pharmaceutical University Osaka Japan

**Keywords:** activities of daily living, endoscopic resection, functional status, gastrointestinal neoplasms, geriatric assessment

## Abstract

**Objectives:**

Endoscopic resection (ER) is used in older patients to treat upper gastrointestinal (UGI) neoplasms due to minimal invasiveness and excellent short‐term therapeutic outcomes. However, its impact on functional outcomes remains unclear. This study aimed to identify functional‐decline predictors post‐ER in older patients.

**Methods:**

This prospective, single‐center cohort study included patients aged ≥75 years undergoing ER for UGI neoplasms between April 2017 and December 2021. Cognitive and physical functions were assessed using the Mini‐Mental State Examination (MMSE) and Instrumental Activities of Daily Living (IADL) scales, before and 6–9 months post‐ER. Functional decline was defined as a decrease of ≥3 points in MMSE or ≥1 point in IADL. Scores of geriatric assessment tools, including the Geriatric 8 (G8), Vulnerable Elders Survey‐13 (VES‐13), Flemish Triage Risk Screening Tool, and Mini‐Cognitive Assessment Instrument (Mini‐Cog), were evaluated as potential functional‐decline predictors.

**Results:**

Of 202 patients, 37 (18.3%) experienced functional decline post‐ER. In multivariate analysis, poor G8 scores (≤14) were independent risk factors for functional decline (odds ratio: 2.64, 95% confidence interval: 1.02–6.84, *p* = 0.0461). Functional‐decline incidence gradually increased as G8 scores decreased (*p* = 0.0086, trend test).

**Conclusions:**

Preoperative G8 scores may serve as functional‐decline predictors in older patients undergoing ER for UGI neoplasms. A preoperative G8 assessment could facilitate risk‐based treatment decisions from the perspective of functional outcomes in this vulnerable population.

## 1 Introduction

With the aging population, the detection of upper gastrointestinal (UGI) neoplasms is increasing, leading to more frequent endoscopic resections (ERs) in older individuals [1, 2, 3]. For ER in older patients, cancer prognosis and potential changes in overall functional status, including physical and cognitive functions, should be considered, given their higher comorbidity risk, reduced activities of daily living (ADL), and decreased quality of life (QOL). Although ER is minimally invasive, functional decline in older patients may result from factors other than the procedure itself. Hospitalization‐related stressors, including fasting, bed rest, and reduced activity, may contribute to decreased muscle strength and cognitive function in frail older individuals. In elderly patients, even modest declines in ADL or cognitive function can substantially affect independence and QOL, sometimes more so than changes in survival. Although survival remains an important endpoint, functional‐decline detection and prevention are critical for maintaining autonomy and well‐being. Furthermore, such declines may not be readily apparent without targeted assessments, as illustrated by cases of reduced appetite, decreased motivation, or cognitive deterioration post‐ER. Therefore, in decision‐making regarding ER for UGI neoplasms in older patients, objective evaluation methods are needed to predict treatment‐associated decline in overall functional status.

Several tools have been investigated to predict overall prognosis in older patients with UGI cancer, including the American Society of Anesthesiologists Physical Status (ASA‐PS) [4, 5], Charlson Comorbidity Index (CCI) [6, 7, 8], Prognostic Nutritional Index (PNI) [9], and Neutrophil‐to‐Lymphocyte Ratio [10]. However, these tools have been evaluated in retrospective studies; no consensus exists on their effectiveness in assessing the functional status of older patients. Recently, geriatric assessment (GA) has been developed to evaluate the physical, cognitive, and social functioning of older patients by assessing frailty and functional‐decline risks. Particularly, screening tools for multidimensional health problems, such as Geriatric 8 (G8) [11], Vulnerable Elders Survey‐13 (VES‐13) [12], the Flemish version of the Triage Risk Screening Tool (f‐TRST) [13, 14], and Mini‐Cognitive Assessment Instrument (Mini‐Cog) [15], can be implemented in a short time and are expected to be used in daily clinical practice.

Although these tools can predict long‐term functional decline post‐surgery or chemotherapy in older patients with cancers [16, 17, 18, 19, 20, 21, 22], no studies have evaluated their usefulness in the ER for UGI neoplasms. Thus, in this prospective study, we aimed to identify screening tools for GA that are useful for predicting functional changes in older patients post‐ER of early UGI neoplasms.

## 2 Methods

### 2.1 Study Design and Population

This single‐center prospective cohort study was conducted at our institution (Osaka International Cancer Institute, Osaka, Japan) between April 2017 and December 2021. The study population consisted of patients aged ≥75 years who underwent ER for UGI (esophageal, gastric, or duodenal) neoplasms. The eligibility criteria were (1) diagnosis of UGI neoplasms on pre‐ER endoscopy and biopsy; (2) age ≥75 years at the time of registration; and (3) provision of written informed consent. The exclusion criteria were (1) refusal to participate in this trial; (2) failure to undergo ER; (3) receipt of additional treatment (i.e., surgery, chemotherapy, or radiotherapy) post‐ER, to isolate the impact of ER on outcomes; or (4) absence from the scheduled 6–9 months follow‐up post‐ER.

The study protocol was approved by the Institutional Review Board and Ethics Committee (approval no. 1703315280–4) and was conducted in accordance with the Declaration of Helsinki. It was registered in the University Hospital Medical Information Network Clinical Trials Registry (UMIN000026216). All patients provided written informed consent prior to participation.

### 2.2 Study Overview

**FIGURE 1:**
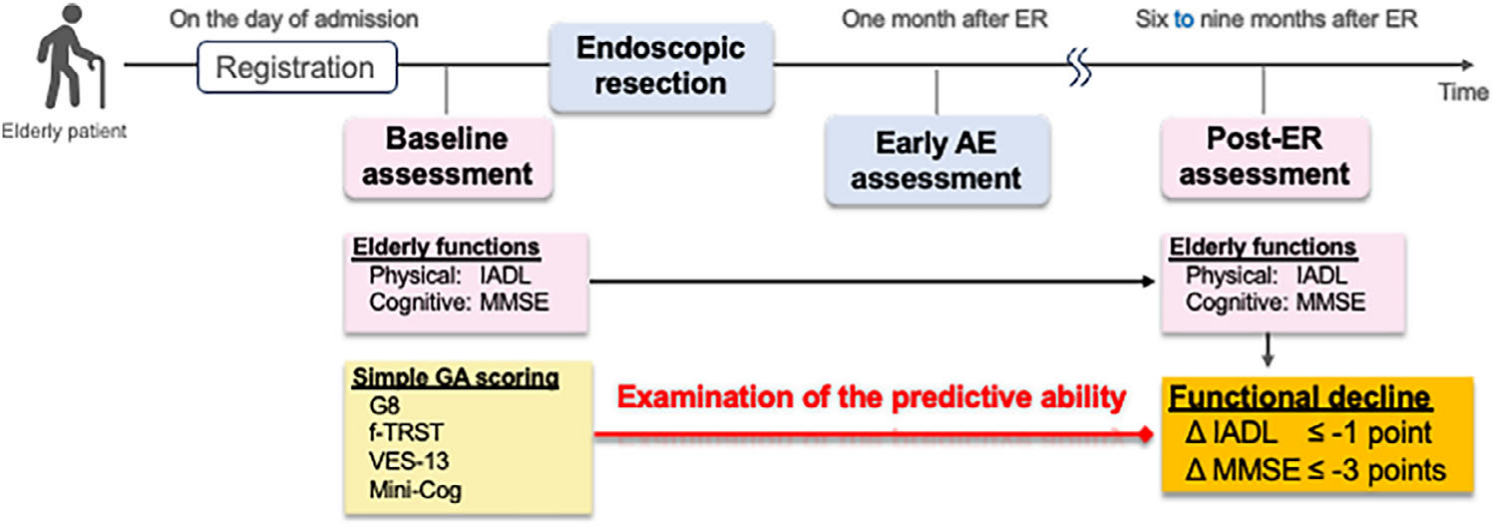
Overall flow of the study assessing the predictive ability of geriatric assessment (GA) tools for functional decline in older patients undergoing endoscopic resection (ER) for upper gastrointestinal (UGI) neoplasms. Baseline assessments of physical and cognitive functions, using IADL and MMSE, along with GA tools (G8, f‐TRST, VES‐13, Mini‐Cog), were performed on the day of admission. Early adverse events (AEs) were assessed 1 month post‐ER. Functional decline, defined as a decrease of ≥1 point in IADL or ≥3 points in MMSE, was assessed 6–9 months post‐ER to evaluate the predictive ability of the GA tools VES‐13, Vulnerable Elders Survey‐13; f‐TRST, Flemish Triage Risk Screening Tool; Mini‐Cog, Mini‐Cognitive Assessment Instrument; G8, Geriatric 8 Screening Tool.

The study consisted of three steps: (1) baseline assessment, (2) ER and early adverse event (AE) assessment, and (3) post‐ER assessment (Figure 1).

#### 2.2.1 Baseline Assessment

At the time of study registration (hospital admission for ER), patient characteristics, including age, sex, and the organ of the target lesion, were recorded. Pre‐ER physical and cognitive functions of the patients were evaluated using the Instrumental Activities of Daily Living (IADL, maximum score 8) and the Mini‐Mental State Examination (MMSE, maximum score 30). In addition to performance status (PS) and CCI, simple GA tools, including the G8, VES‐13, f‐TRST, and Mini‐Cog, were administered before treatment as potential functional‐decline predictors post‐ER. These GA tools can be completed within minutes: G8 focuses on nutritional status [11], VES‐13 on physical function [12], f‐TRST on triage in the acute phase [14], and Mini‐Cog on cognitive function [15].

#### 2.2.2 ER and Early AE Assessment

For each target lesion, the choice between endoscopic mucosal resection (EMR) and endoscopic submucosal dissection (ESD) was determined by the endoscopist. Both procedures were performed following the standardized clinical pathway at our institution. Postoperatively, patients fasted on the day after the procedure and were gradually reintroduced to a soft diet. In the absence of significant AEs, discharge typically occurred on postoperative days 4–5. ER‐related outcomes, including tumor location, tumor size, tumor morphology, clinical depth of the lesion, resection method (EMR or ESD), and histology of the resected specimen, were recorded. Early AEs occurring within 1 month, such as postponement of discharge and readmission within 2 weeks, were also documented; these included events that could not be definitively ruled out as ER‐related.

Based on the pathological diagnosis post‐ER, additional treatments were administered as necessary according to our clinical practice; such cases were excluded from the study population.

#### 2.2.3 Post‐ER Assessment

Subsequently, 6–9 months post‐ER, physical and cognitive functional decline was evaluated during outpatient visits. The follow‐up interval was set at 6–9 months post‐ER, rather than at a fixed time point, to account for practical issues such as geographical distance or the burden of family accompaniment, which sometimes made timely hospital visits difficult for elderly patients. As in the pre‐ER assessment, functional evaluation was conducted using the IADL and MMSE. In addition to declines in physical and cognitive function compared to pretreatment levels, post‐ER AEs also included any hospitalization possibly related to ER, as assessed through interviews and chart review. For patients who missed follow‐up visits at 6–9 months post‐ER, telephone interviews were conducted to confirm survival status and health changes, including weight loss, reduced food intake, decreased ADLs or PS, cognitive decline, new illness, and hospitalization.

### 2.3 Outcome Measures and Definitions

The primary endpoint was the incidence of decline in physical and cognitive functions post‐ER compared to pre‐ER levels. Decline was defined as (1) a decrease of ≥1 point (1.0 standard deviation [SD]) in IADL or (2) a decrease of ≥3 points (1.0 SD) in MMSE. As no universal cutoffs exist for defining functional decline in MMSE and IADL, we adopted a data‐driven approach. Specifically, decline was defined based on 1.0 SD of the baseline distribution in our study population, which was considered to represent a clinically meaningful change in functional status. This is supported by previous studies showing that each 1‐point increase in IADL score was associated with a 16% reduction in mortality risk among patients with heart failure (hazard ratio, 0.84; 95% confidence interval [95%CI], 0.71–0.99) [23] and that a decrease of ≥3 points in MMSE can serve as a marker of clinically relevant cognitive decline in longitudinal studies [24]. The secondary endpoint was the incidence of early AEs, such as endoscopy‐related postponement of discharge (>3 days beyond schedule) or readmission within 2 weeks.

### 2.4 Statistical Analyses

Quantitative variables are presented using median values with interquartile ranges (IQR) or mean values with SD, as appropriate. Qualitative variables are presented using counts and proportions. Continuous GA scores were categorized using cutoffs from previous reports [11, 25, 26, 27]. Age ≥80 years, PS ≥2, CCI ≥2, G8 ≤14, f‐TRST ≥1, VES‐13 ≥3, and Mini‐Cog ≤2 were classified as “poor.”

Additionally, for the G8 score, receiver operating characteristic (ROC) curve analysis was performed to determine the optimal cutoff value to predict functional decline. The area under the curve (AUC), sensitivity, and specificity were calculated; the optimal cutoff was evaluated and compared with the conventionally used threshold of 14. Associations between qualitative variables were tested using the Chi‐square or Fisher's exact test. The association between pretreatment GA tool scores and the primary outcome was examined using univariate analysis. The univariate analysis included age, sex, PS, CCI, G8 score, f‐TRST score, VES‐13 score, Mini‐Cog score, early AEs, tumor location, and ER method (EMR or ESD). Multivariate analysis was performed using variables significant in univariate analysis. The Cochran‐Armitage trend test was used to examine relationships between categorical variables and event occurrence. Statistical significance was set at *p* < 0.05. All statistical analyses were conducted using EZR, version 4.2.2 (Saitama Medical Center, Jichi Medical University, Japan).

To assess potential effects of follow‐up interval variation on functional‐decline incidence, patients were classified according to the follow‐up interval: ≤200 days and >200 days post‐ER. Functional‐decline incidence was compared between these groups.

To evaluate the potential impact of missing follow‐up, a sensitivity analysis was performed under two extreme assumptions: (1) all patients lost to follow‐up experienced functional decline; (2) none experienced functional decline. Statistical significance was reassessed under each assumption.

## 3 Results

**FIGURE 2:**
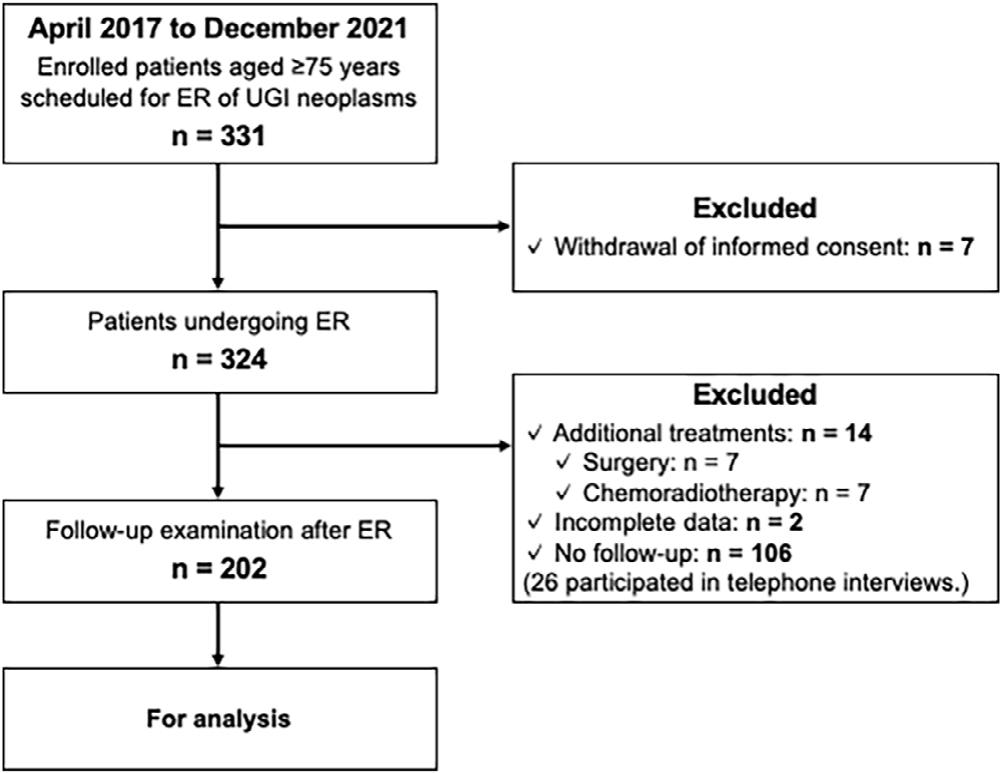
Flowchart of the study population selection. ER, Endoscopic Resection; UGI, Upper Gastrointestinal.

Between April 2017 and December 2021, 331 patients aged ≥75 years scheduled for ER for UGI neoplasms were prospectively enrolled; seven withdrew consent. Among the remaining 324 patients who underwent ER, 14 were excluded owing to additional treatments (surgery, *n* = 7; chemoradiotherapy, *n* = 7) and two owing to incomplete data; 106 patients did not attend outpatient follow‐up, leaving 202 for the final analysis (Figure 2).

Baseline characteristics are presented in Table 1. The median age was 80 (78–83) years: patients aged 75–79 years, 81 (40.1%); 80–84 years, 82 (40.6%); and ≥85 years, 39 (19.3%). Men comprised 84.7% (171/202) of the cohort. The most common tumor location was the stomach (122/202, 60.4%), followed by the esophagus (68/202, 33.7%) and duodenum (13/202, 6.4%). ESD was performed in 171 patients (84.7%) and EMR in 31 patients (15.3%). One patient had both gastric and esophageal lesions. Baseline mean MMSE and IADL scores were 27.0 (2.95) and 5.29 (1.30), respectively. Mean GA scores were as follows: G8, 13.37 (2.16); f‐TRST, 0.99 (0.92); VES‐13, 2.01 (1.51); and Mini‐Cog, 3.85 (1.18).

**TABLE 1:** Pre‐endoscopic resection (ER) characteristics of enrolled patients.

Patients (*n* = 202)	
Age, median (IQR), years	81 (78‐83)
75–79, *n* (%)	81 (40.1)
80–84, *n* (%)	82 (40.6)
≥85, *n* (%)	39 (19.3)
Sex, *n*	
Male/Female	171/31
PS, *n* (%)	
0	193 (95.5)
≥1	9 (4.5)
CCI, *n* (%)	
0	89 (44.1)
1	54 (26.7)
≥2	59 (29.2)
Location, *n* (%)	
Esophagus	68 (33.7)
Stomach	122 (60.4)
Duodenum	13 (6.4)
ER method, *n* (%)	
ESD	171 (84.7)
EMR	31 (15.3)
Older people's functions, mean ± SD, %	
MMSE	27.0 ± 2.95
IADL	5.29 ± 1.30
GA scores, mean ± SD, %	
G8	13.37 ± 2.16
f‐TRST	0.99 ± 0.92
VES‐13	2.01 ± 1.51
Mini‐Cog	3.85 ± 1.18

Abbreviations: CCI, Charlson Comorbidity Index; EMR, Endoscopic Mucosal Resection; ER, Endoscopic Resection; ESD, Endoscopic Submucosal Dissection; f‐TRST, Flemish Triage Risk Screening Tool; GA, Geriatric Assessment; G8, Geriatric 8 Screening Tool; IADL, Instrumental Activities of Daily Living; IQR, Interquartile Range; Mini‐Cog, Mini‐Cognitive Assessment Instrument; MMSE, Mini‐Mental State Examination; PS, performance status; SD, Standard Deviation; VES‐13, Vulnerable Elders Survey‐13.

Study outcomes are summarized in Table 2. Functional decline, the primary endpoint, occurred in 37 patients, including 25 with decreases ≥3 points in MMSE and 15 with decreases ≥1 point in IADL. When stratified by follow‐up interval, functional‐decline incidence was 18.4% (33/179) and 17.4% (4/23) in patients followed up for ≤200 days and >200 days, respectively, with no significant differences between groups (Table 3). Early AEs occurred in 12 patients, including nine delayed discharges and three rehospitalizations within 2 weeks.

**TABLE 2:** Functional decline and early adverse events in patients undergoing endoscopic resection.

All patients (*n* = 202)	
Functional decline, *n* (%)	37 (18.3)
MMSE decrease (>3 points)	25 (12.4)
IADL decrease (>1 point)	15 (7.4)
Early AE, *n* (%)	12 (5.9)
Hospitalization postponement	9 (4.5)
Readmission in 2 weeks	3 (1.5)

Abbreviations: AE, Adverse Event; IADL, Instrumental Activities of Daily Living; MMSE, Mini‐Mental State Examination.

**TABLE 3:** Functional‐decline incidence according to the follow‐up interval (days).

Follow‐up interval (days)	Functional decline, *n* (%)
≤200	33/179 (18.4%)
>200	4/23 (17.4%)

**FIGURE 3:**
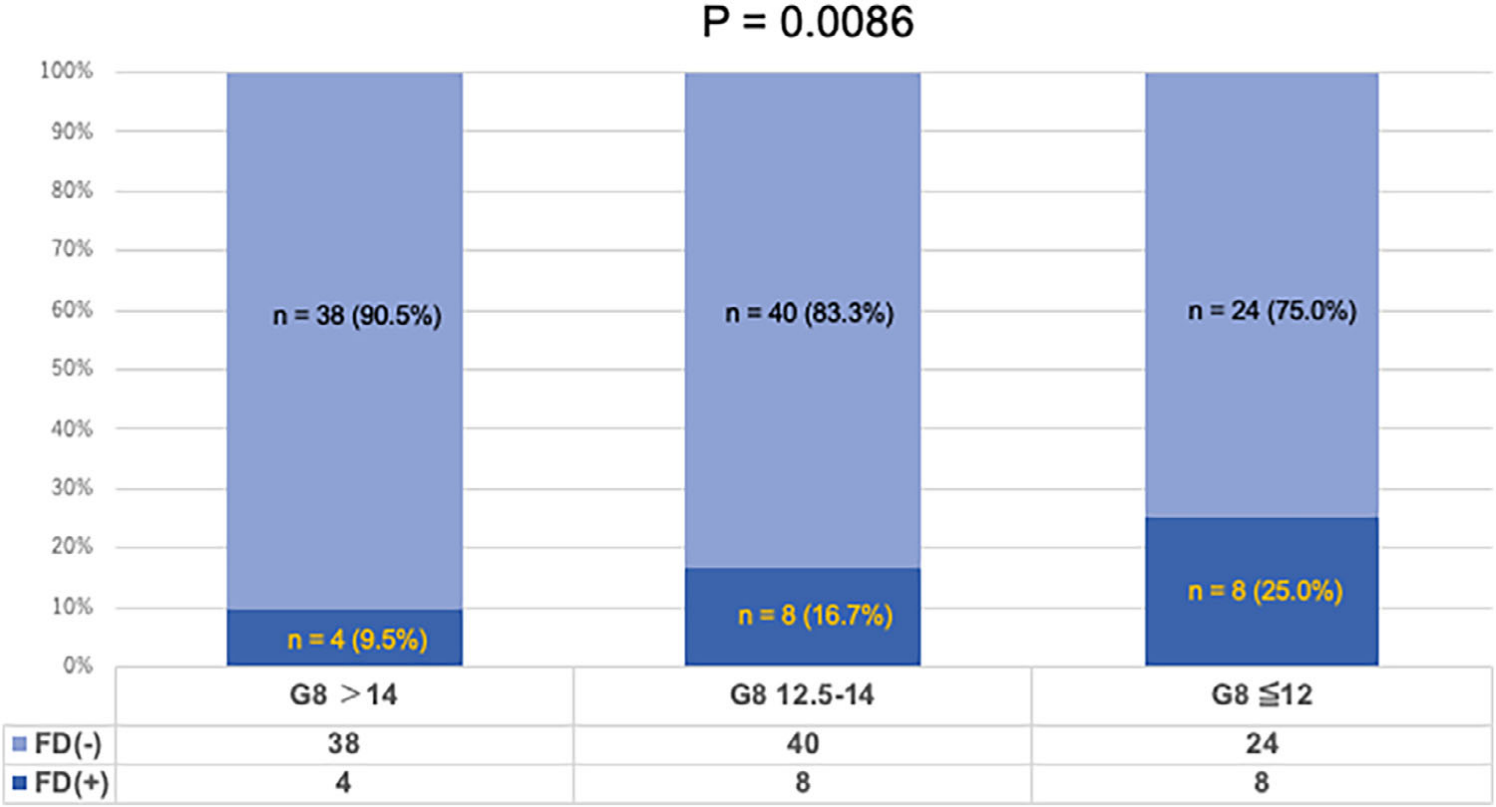
Functional decline based on G8 scores (Cochran‐Armitage Trend Test). ER, Endoscopic Resection; UGI, Upper Gastrointestinal; FD, Functional Decline; G8, Geriatric 8 Screening Tool.

**FIGURE 4:**
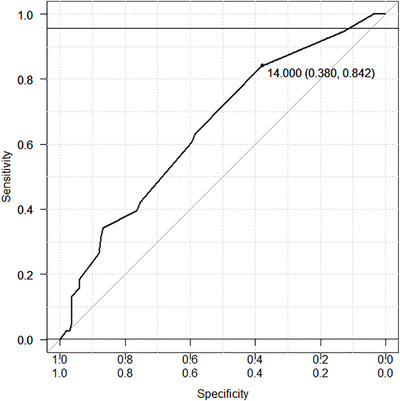
Receiver operating characteristic (ROC) curve for G8 scores in predicting functional decline post‐endoscopic resection. ROC analysis yielded an area under the curve (AUC) of 0.653 (95%CI, 0.559–0.748). The optimal cutoff value was 14, with a sensitivity of 84.2% and specificity of 38.0%.

Clinical features were compared between patients with and without functional decline post‐ER. In univariate analysis, a poor preoperative G8 score (≤14) was a functional‐decline predictor (31/134 [23.1%] vs. 6/68 [8.8%]; *p* = 0.0126). Other factors, including PS, CCI, f‐TRST scores, VES‐13 scores, early AEs, tumor location, and ER method, were not significantly associated with functional decline (Table 4). Multivariate analysis, including age, sex, and the G8 score, confirmed that G8 ≤14 was an independent functional‐decline predictor post‐ER (odds ratio: 2.64, 95%CI: 1.02–6.84, p = 0.0461). Furthermore, patients were divided into three groups based on preoperative G8 scores: ≤12, 12.5–14, and >14. Functional‐decline incidence increased as preoperative G8 scores decreased, and this trend was statistically significant (*p* = 0.0086 in the Cochran‐Armitage trend test) (Figure 3). Additionally, ROC curve analysis of G8 scores for predicting functional decline yielded an AUC of 0.653 (95%CI: 0.559–0.748). The optimal cutoff was 14, with 84.2% sensitivity and 38.0% specificity (Figure 4).

**TABLE 4:** Univariate and multivariate analyses of functional‐decline predictors following endoscopic resection.

	Functional decline (−) *n* = 165		Functional decline (+) *n* = 37		Univariate analysis	Multivariate analysis		
					*p*‐value^*^	OR	95%CI	*p*‐value^**^
Age, median (IQR), years	81	(78–83)	80	(78–83)	0.0404			0.112
75–79, *n*	72		9			Reference		
≥80, *n*	93		28			1.97	0.854–4.57	
Sex, *n*					0.806			0.700
Male	140		31			0.821	0.302–2.24	
Female	25		6			Reference		
PS, *n*					1			
≥2 (Poor)	2		0					
<2 (Good)	163		37					
G8, *n*					0.0126			0.0461
≤14 (Poor)	103		31			2.64	1.02–6.84	
>14 (Good)	62		6			Reference		
CCI, *n*					0.847			
≥2 (Poor)	51		12					
<2 (Good)	114		25					
Mini‐Cog, *n*					0.207			
≤2 (Poor)	22		8					
>2 (Good)	143		29					
f‐TRST, *n*					0.252			
≥1 (Poor)	108		28					
<1 (Good)	57		9					
VES‐13, *n*					0.144			
≥3 (Poor)	38		13					
<3 (Good)	127		24					
Early AE, *n*					0.463			
(+)	9		3					
(−)	156		34					
Location, *n*					0.704			
Esophagus	54		14					
Stomach	102		20					
Duodenum	10		3					
ER method, *n* (%)					0.806			
EMR	25		6					
ESD	140		31					

Abbreviations: 95%CI, 95% Confidence Interval; AE, Adverse Event; CCI, Charlson Comorbidity Index; EMR, Endoscopic Mucosal Resection; ER, endoscopic resection; ESD, Endoscopic Submucosal Dissection; f‐TRST, Flemish Triage Risk Screening Tool; G8, Geriatric 8 Screening Tool; IQR, Interquartile Range; Mini‐Cog, Mini‐Cognitive Assessment Instrument; OR, Odds Ratio; PS, performance status; VES‐13, Vulnerable Elders Survey‐13.

In the sensitivity analysis, assuming that all patients lost to follow‐up experienced functional decline, the association between G8≤14 and functional decline remained significant (odds ratio [OR]: 2.04, 95%CI: 1.21–3.45, *p* = 0.008). In contrast, on assuming that no patients lost to follow‐up experienced functional decline, the association was attenuated and not statistically significant (OR: 2.10, 95%CI: 0.83–5.35, *p* = 0.118) (Table 5). Thus, loss to follow‐up may have influenced findings; however, baseline characteristics were generally comparable between patients lost to follow‐up and those who completed follow‐up (Table 6).

**TABLE 5:** Sensitivity analysis for the association of G8 score (≤14 vs. >14) with functional decline under different assumptions for patients lost to follow‐up.

Assumption for patients lost to follow‐up	Functional decline, *n* (%)	OR (95%CI) for G8 ≤14	*p*‐Value
Base case (patients with follow‐up only)	37/202 (18.3%)	2.64 (1.02–6.84)	0.046
All lost to follow‐up = functional decline	143/308 (46.4%)	2.04 (1.21–3.45)	0.008
None lost to follow‐up = functional decline	37/308 (12.0%)	2.10 (0.83–5.35)	0.118

Abbreviations: 95%CI, 95% confidence interval; G8, Geriatric 8 Screening Tool; OR, odds ratio.

**TABLE 6:** Baseline characteristics of patients with and without follow‐up.

	Patients with follow‐up (*n* = 202)	Patients without follow‐up (*n* = 106)
Age, median (IQR), years	81 (78–83)	80 (77–84)
75–79, *n* (%)	81 (40.1)	44 (41.5)
80–84, *n* (%)	82 (40.6)	42 (39.6)
≥85, *n* (%)	39 (19.3)	20 (18.9)
Sex, *n* (%)		
Male/Female	171/31	73 (68.9)/35 (31.1)
PS, *n* (%)		
0	193 (95.5)	102 (96.2)
≥1	9 (4.5)	4 (3.8)
CCI, *n* (%)		
0	89 (44.1)	41 (38.7)
1	54 (26.7)	26 (24.5)
≥2	59 (29.2)	39 (27.4)
Location, *n* (%)		
Esophagus	68 (33.7)	26 (24.5)
Stomach	122 (60.4)	70 (66.0)
Duodenum	13 (6.4)	10 (9.4)
ER method, *n* (%)		
ESD	171 (84.7)	89 (84.0)
EMR	31 (15.3)	17 (16.0)
Elderly functions, mean ± SD, %		
MMSE	27.0 ± 2.95	26.39 ± 3.07
IADL	5.29 ± 1.30	5.71 ± 1.83
GA scores, mean ± SD, %		
G8	13.37 ± 2.16	13.28 ± 1.94
f‐TRST	0.99 ± 0.92	1.42 ± 1.08
VES‐13	2.01 ± 1.51	2.35 ± 2.16
Mini‐Cog	3.85 ± 1.18	4.02 ± 1.09

Abbreviations: CCI, Charlson Comorbidity Index; EMR, Endoscopic Mucosal Resection; ER, Endoscopic Resection; ESD, Endoscopic Submucosal Dissection; f‐TRST, Triage Risk Screening Tool; GA, Geriatric Assessment; G8, Geriatric 8 Screening Tool; IADL, Instrumental Activities of Daily Living; IQR, Interquartile Range; Mini‐Cog, Mini‐Cognitive Assessment Instrument; MMSE, Mini‐Mental State Examination; PS, performance status; SD, Standard Deviation; VES‐13, Vulnerable Elders Survey‐13.

For 106 patients without follow‐up visits, baseline characteristics are detailed in Table S1. The median age was 80 (77–84) years: patients aged 75–79 years, 44 (41.5%); 80–84 years, 42 (39.6%); and ≥85 years, 20 (18.9%). Men comprised 68.9% (73/106) of the cohort. The most common tumor location was the stomach (70/106, 66.0%), followed by the esophagus (26/106, 24.5%) and duodenum (10/106, 9.4%). ESD was performed in 89 patients (84.0%) and EMR in 17 patients (16.0%). Mean MMSE and IADL scores were 26.39 (3.07) and 5.71 (1.83), respectively. Telephone interviews were completed in 26/106 (24.5%); none had died or experienced severe long‐term outcomes. Two patients (12.8%) showed functional decline. One was hospitalized for cerebral infarction and pneumonia, with PS decreasing from 0 to 3. The other was hospitalized for 3 months due to coronavirus disease 2019 (COVID‐19) [28], with PS dropping from 0 to 2 but without cognitive deterioration.

## 4 Discussion

This prospective study evaluated the utility of multiple GA screening tools in predicting functional decline post‐ER in patients aged >75 years. The G8 was the most reliable functional‐decline predictor post‐ER for UGI neoplasms. To our knowledge, this is the first study to demonstrate the effectiveness of a GA screening tool in the context of endoscopic treatment.

The Cochran‐Armitage test showed a higher likelihood of functional decline with lower G8 scores. The G8 is valuable for its simplicity and ability to assess age, physical and cognitive functions, medication use, and nutrition, providing a holistic view of older patients. Unlike conventional decision‐making, relying on age, PS, and comorbidities, the G8 offers an objective assessment of ER readiness. Additionally, the G8 stratifies patients by total scores, not binary responses, and can be completed within 5 min, supporting its clinical application.

A poor G8 score should not automatically preclude treatment but should prompt a comprehensive risk evaluation. Other tools, including VES‐13, f‐TRST, and Mini‐Cog, were not predictive. This is likely because the G8 captures multiple domains, particularly nutrition, whereas VES‐13 and f‐TRST emphasize physical function and Mini‐Cog focuses primarily on cognition, limiting their sensitivity to multifactorial decline. In clinical practice, multidisciplinary input remains important. Herein, the optimal cutoff identified by ROC analysis was 14, consistent with previous findings. Although the mean G8 score (13.37) was slightly lower than reported previously, reflecting the cohort's advanced age and comorbidities, the conventional cutoff performed similarly. Thus, a threshold of 14 may be applied, although re‐evaluation may be required in future studies.

This study has some limitations. First, it was a single‐center study, which may limit the generalizability of the findings. However, the study included >200 patients, providing a robust sample size. Second, a significant number of patients were lost to follow‐up. However, these patients had a slightly higher mean preoperative G8 score (13.7) than those who completed the study (13.1), and none died during the 6–9‐month follow‐up period. Sensitivity analysis showed that the association between G8 ≤14 and functional decline remained significant under the assumption that all patients lost to follow‐up experienced functional decline; the association was attenuated when assuming none experienced decline, suggesting that loss to follow‐up may have influenced results. However, baseline characteristics were comparable between patients with and without follow‐up; the likely reasons for loss included distance and COVID‐19 [28], rather than ER‐related decline. Therefore, loss to follow‐up was considered to have minimal impact on the study results. Third, the follow‐up period of 6–9 months may not have captured long‐term outcomes. This interval was selected to assess short‐term changes in physical and cognitive functions post‐ER, which are considered highly relevant to decision‐making in elderly patients. Although ER is a minimally invasive procedure with low mortality, even short‐term functional decline can substantially impact patient QOL and may influence treatment choices. Longer follow‐up intervals, such as 1–2 years, risk being confounded by age‐related decline, making it challenging to attribute changes directly to ER. Therefore, we focused on the early post‐treatment period, during which functional changes are more likely to be causally related to the intervention. Nonetheless, future studies should evaluate the persistence of decline and its effect on QOL and survival. Additionally, we examined whether follow‐up interval variation within the 6–9‐month window affected functional‐decline incidence by stratifying patients into two groups: ≤200 days and >200 days post‐ER. No significant differences were found between groups; thus, this variation was unlikely to have influenced our primary outcome. Nonetheless, future studies with standardized follow‐up schedules may further minimize potential timing‐related effects. Fourth, this study was an exploratory analysis to identify potential GA tools to predict functional decline post‐ER in older adults; no control cohort without UGI neoplasms was included. Therefore, whether the G8 is predictive beyond ER for UGI neoplasms remains unclear. These findings provide preliminary evidence to support future prospective, multicenter studies with control groups to validate the utility of the G8 and examine long‐term prognosis.

In conclusion, preoperative G8 evaluation is a valuable tool to assess functional‐decline risk in older patients undergoing ER for UGI neoplasms. Its incorporation into clinical practice may facilitate more accurate risk assessment and better‐informed treatment decisions for older patients considering endoscopic procedures.

## Author Contributions

**Conceptualization and design**: Yuki Okubo, Takahiro Inoue, Shunsuke Yoshii, Masamichi Arao, Taro Iwatsubo, Hiroko Nakahira, Katsunori Matsueda, and Ryu Ishihara. **Analysis and interpretation of the data**: Yuki Okubo, Takahiro Inoue, Shunsuke Yoshii, and Ryu Ishihara. **Drafting of the article**: Yuki Okubo, Takahiro Inoue, Shunsuke Yoshii, and Ryu Ishihara. **Critical revision of the article for important intellectual content**: Shunsuke Yoshii and Ryu Ishihara. **Final approval of the article**: Yuki Okubo, Takahiro Inoue, Shunsuke Yoshii, Masamichi Arao, Taro Iwatsubo, Hiroko Nakahira, Katsunori Matsueda, Minoru Kato, Satoki Shichijo, Takashi Kanesaka, Sachiko Yamamoto, Koji Higashino, Noriya Uedo, Tomoki Michida, and Ryu Ishihara.

## Conflicts of Interest

The authors declare no conflicts of interest.

## Ethics Statement

**Approval of the research protocol by an Institutional Reviewer Board**: All procedures followed were in accordance with the ethical standards of the Institutional Review Board and Ethics Committee (approval no. 1703315280–4) and with the Helsinki Declaration of 1964 and later versions.

## Informed Consent

Informed consent or a substitute for it was obtained from all patients for being included in the study.

## Clinical Trial Registration

The study was registered in the University Hospital Medical Information Network Clinical Trials Registry (UMIN000026216).

## Supporting information




**Table S1**: Characteristics of the patients who did not attend follow‐up visits.

## Data Availability

The authors have nothing to report.
