# Supplementary material for: Geriatric 8 Score Predicts Functional Decline After Endoscopic Resection for Upper Gastrointestinal Neoplasms in Older Adults: A Prospective Cohort Study
Source: DEN Open. 2025 Oct 18;6(1):e70226. doi: 10.1002/deo2.70226 (PMC12535265; doi:10.1002/deo2.70226)
Supplement: Supplementary file 1 — Table S1: Characteristics of the patients who did not attend follow‐up visits. [file DEO2-6-e70226-s001.docx]

Supplementary Table 1. Characteristics of the patients who did not attend follow-up visits.

| Patients (n = 106) | |
| --- | --- |
| Age, median (IQR), years  75–79, n (%)  80–84, n (%)  ≥85, n (%) | 80 (77-84)  44 (41.5)  42 (39.6)  20 (18.9) |
| Sex, n (%)  Male/Female | 73 (68.9)/35 (31.1) |
| PS, n (%)  0  ≥1 | 102 (96.2)  4 (3.8) |
| CCI, n (%)  0  1  ≥2 | 41 (38.7)  26 (24.5)  39 (27.4) |
| Location, n (%)  Esophagus  Stomach  Duodenum | 26 (24.5)  70 (66.0)  10 (9.4) |
| ER method, n (%)  ESD  EMR | 89 (84.0)  17 (16.0) |
| Elderly functions, mean ± SD, %  MMSE  IADL | 26.39 ± 3.07  5.71 ± 1.83 |
| GA scores, mean ± SD, %  G8  f-TRST  VES-13  Mini-cog | 13.28 ± 1.94  1.42 ± 1.08  2.35 ± 2.16  4.02 ± 1.09 |

ER, Endoscopic Resection; IQR, Interquartile Range; ESD, Endoscopic Submucosal Dissection; EMR, Endoscopic Mucosal Resection; MMSE, Mini-Mental State Examination; IADL, Instrumental Activities of Daily Living; GA, Geriatric Assessment; G8, Geriatric 8 Screening Tool; f-TRST, Triage Risk Screening Tool; VES-13, Vulnerable Elders Survey-13; SD, Standard Deviation.
